# Case report: Eosinophilic fasciitis induced by pembrolizumab with high FDG uptake on ^18^F-FDG-PET/CT

**DOI:** 10.3389/fmed.2022.1078560

**Published:** 2022-12-20

**Authors:** Karim Amrane, Coline Le Meur, Philippe Thuillier, Pierre Alemany, Clémence Niel, David Renault, Ronan Abgral

**Affiliations:** ^1^Department of Oncology, Centre Hospitalier des Pays de Morlaix, Morlaix, France; ^2^Institut National de la Santé et de la Recherche Médicale UMR 1227, Lymphocytes B et Autoimmunité, Univ. Brest, Institut National de la Santé et de la Recherche Médicale, LabEx IGO, Brest, France; ^3^Institut National de la Santé et de la Recherche Médicale UMR 1304 GETBO, University of Western Brittany, Brest, France; ^4^Department of Endocrinology, University Hospital of Brest, Brest, France; ^5^Department of Pathology, Ouest Pathologie, Brest, France; ^6^Department of Pneumology, Centre Hospitalier des Pays de Morlaix, Morlaix, France; ^7^Department of Nuclear Medicine, University Hospital of Brest, Brest, France

**Keywords:** NSCLC, eosinophilic fasciitis, ^18^F-FDG-PET/CT, pembrolizumab, ICI

## Abstract

Eosinophilic fasciitis (EF) is a rare connective tissue disorder causing inflammation and fibrosing of fascia. In this study, we present a very rare case of an immune checkpoint inhibitor (ICI)-induced EF revealed by ^18^F-fluorodesoxyglucose positron emission tomography (FDG-PET/CT) 20 months after the initiation of Pembrolizumab treatment of a relapsed non-small cell lung cancer (NSCLC). This study presents a 52-year-old Caucasian woman clinically presenting asthenia, inflammatory muscle, and joint pain associated with subcutaneous nodules and symmetrical edema of the lower limbs. Iterative ^18^FDG-PET/CT scans allow us to guide the therapeutic strategy due to this atypical ICI adverse event.

## Introduction

Eosinophilic fasciitis (EF) is a scleroderma-like syndrome, a rare connective tissue disorder causing inflammation and fibrosing of fascia with variable clinical manifestations that are occurring spontaneously (1) or being induced by immune checkpoint inhibitors (ICIs), usually late after treatment initiation (2, 3). EF is frequently progressive and associated with severe and incapacitating joint flexion contractures (1).

## Case description

**FIGURE 1:**
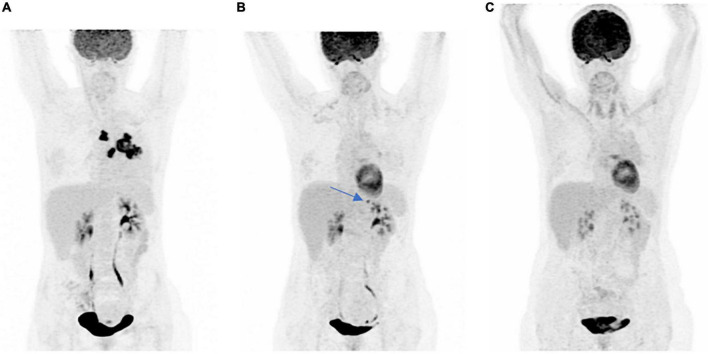
**(A)** FDG-PET MIP: initial staging, **(B)** FDG-PET MIP: left adrenal relapse, arrow, and **(C)** FDG-PET MIP: complete response with diffuse immune hypothyroidism.

We present a 52-year-old woman with a previous medical history of locally advanced non-small-cell lung carcinoma (NSCLC) (Figure 1A–FDG-PET MIP: initial staging) with the PD-L1 expression level of 5%, treated by radiochemotherapy. After 2 years, she presented an adrenal relapse (Figure 1B–FDG-PET MIP: left adrenal relapse, arrow), which was treated with stereotactic radiotherapy and pembrolizumab (4), achieving a complete response (CR) after 15 months of treatment (20 cycles) [Figure 1C–FDG-PET MIP: complete response with diffuse immune hypothyroidism corresponding to immune-induced hypothyroidism by ICI (CTCAE 5.0 grade 2) that had been treated by supplementation].

**FIGURE 2:**
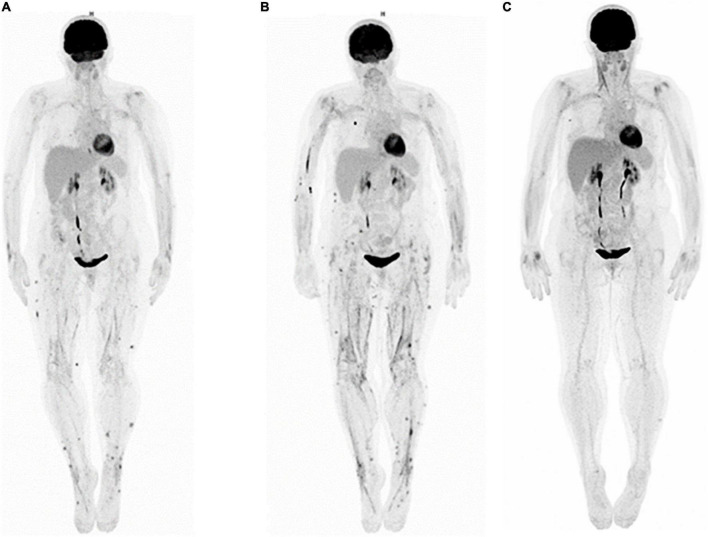
Whole-body FDG-PET MIP: **(A)** appearance of subcutaneous and fascial hypermetabolism, **(B)** increase of subcutaneous and fascial hypermetabolism, and **(C)** disappearance of subcutaneous and fascial hypermetabolism, after corticotherapy.

**FIGURE 3:**
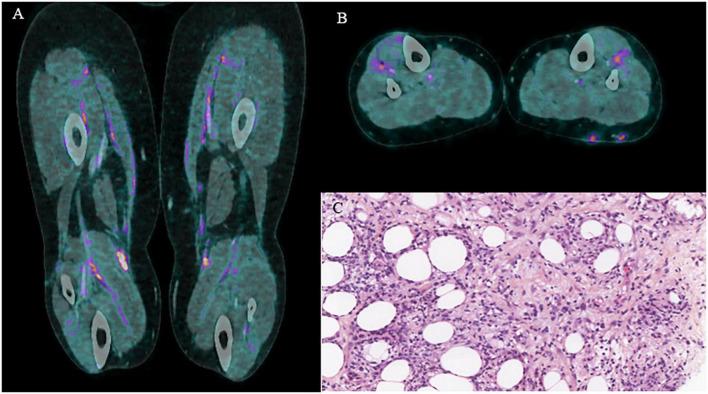
**(A)** Coronal l FDG PET-CT. **(B)** Axial FDG PET-CT. **(C)** Histologic feature of eosinophilic fasciitis.

After six cycles of treatment with pembrolizumab, a 2-deoxy-2-[18F] fluoro-D-glucose positron emission tomography-computed tomography (^18^F-FDG-PET/CT) evaluation showed the persistence of CR, however, revealing the appearance of subcutaneous and fascial hypermetabolism (Figure 2A) without any morphological or clinical translation except a transitory elevation of eosinophil count on biological analysis. This asymptomatic state did not justify the introduction of therapy. After four cycles of treatment with Pembrolizumab, the patient complained of CTCAE 5.0 grade 2 asthenia, inflammatory muscle, and joint pain associated with subcutaneous nodules and symmetrical edema of the lower limbs. The blood test revealed a slightly elevated CRP of 20.5 mg/L, with normal levels of cortisol and TSH. A new evaluation by FDG-PET/CT was performed, showing no cancer progression but an increase in the number and intensity of hypermetabolic subcutaneous nodules and muscle fascia (limbs, paravertebral) lesions and the appearance of diffuse tracer uptake on the synovial walls of both knees (Figure 2B). The SUV_*max*_ on fascia was 12.3 and on subcutaneous nodules was 9.5. To explore these hypermetabolic findings (Figures 3A, B coronal and axial FDG PET-CT views), a subcutaneous nodule biopsy was performed and histologic analysis revealed panniculitis with septal inflammation. This septal infiltrate was mostly composed of lymphocytes and few histiocytes; vessels were well seen, without vasculitis (Figure 3C HES x40).

The suspicion of EF seemed obvious given the isotopic presentation and confirmation of the subcutaneous nodule lymphocytic infiltration (5). Both autoantibody assay (antinuclear antibodies, rheumatoid factors, anti-cyclic citrullinated peptide, and anti-Scl70) and electroneuromyogram were negatives, ruling out differential diagnoses such as scleroderma and other scleroderma-like syndromes (6).

The management of this disease consisted of discontinuing Pembrolizumab as it was responsible for this immune-induced adverse event and starting corticosteroid therapy (CST) at 0.5mg/kg in decreasing doses for 3 months for their immunomodulatory effect (7), resulting in resolution of symptoms and reduction and then disappearance of subcutaneous and fascial hypermetabolisms (Figure 2C FDG-PET MIP).

## Discussion

Interestingly, to the best of our knowledge, we found that there are only 16 cases of ICI-induced EF in the literature and none in major therapeutic trials. Among these 16 cases, 50% (8/16) were in CR at the time of onset of this adverse event and 25% (4/16) were in progression disease, mainly in urothelial and renal tumors. Only 12.5% (2/16) were fortuitously revealed by FDG-PET/CT, none in the case of lung cancer (6, 8). The analysis of 16 reported cases shows that the time of onset is late following the initiation of ICI, ranging from 8 to 15 months, except for 2 clinical cases where the time of onset was 3 months. In our case, the time of onset was late since it was 19 months, which is consistent with the trend found in the literature.

Although magnetic resonance imaging (MRI) seems to have a better diagnostic performance than FDG-PET/CT due to its high sensitivity for soft tissue analysis that can show thickening of the muscular fascias in high signal intensity (9, 10), this examination is only performed in the case of diagnostic suspicion and mostly after the onset of symptoms. However, ^18^F-FDG-PET/CT has the advantage of being a whole-body examination for which the additional acquisition of images on the limbs is slightly longer (1–2 min) and does not reach more radiation exposure for the patient. In addition to the assessment of tumor response to ICI, ^18^F-FDG-PET/CT is useful for subclinical detection of fascia tracer uptake, allowing early treatment of EF (11) and also remains an interesting tool for its multifocal involvement evaluation (12, 13).

This case of ICI-induced EF suggests the usefulness of consistently performing a whole-body ^18^F-FDG-PET/CT examination in a patient treated by immunotherapy for not overlooking subcutaneous nodules and fascia FDG uptakes that could guide the early management of this adverse event.

## Ethics statement

Ethical review and approval was not required for the study on human participants in accordance with the local legislation and institutional requirements. Written informed consent from the patients/participants OR patients/participants legal guardian/next of kin was not required to participate in this study in accordance with the national legislation and the institutional requirements.

## Author contributions

KA and RA provided the details of the patient and provided an initial draft of the submission. CN and DR provided the details of the patient. PT, PA, and RA provided the images and image analysis and helped in drafting the initial submission. CL helped in drafting the initial submission. All authors contributed to the article and approved the submitted version.

## References

[1] Shulman LE. Diffuse fasciitis with hypergammaglobulinemia and eosinophilia: a new syndrome? *J Rheumatol.* (1984) 11:569–70. 6542592

[2] Khoja L Maurice C Chappell M MacMillan L Al-Habeeb AS Al-Faraidy N Eosinophilic fasciitis and acute encephalopathy toxicity from pembrolizumab treatment of a patient with metastatic melanoma. *Cancer Immunol Res.* (2016) 4:175–8. 10.1158/2326-6066.CIR-15-0186 26822024

[3] Bronstein Y Ng CS Hwu P Hwu WJ. Radiologic manifestations of immune-related adverse events in patients with metastatic melanoma undergoing anti-CTLA-4 antibody therapy. *AJR Am J Roentgenol.* (2011) 197:W992–1000. 22109345 10.2214/AJR.10.6198

[4] Herbst RS Baas P Kim D-W Kim DW Felip E Pérez-Gracia JL Pembrolizumab versus docetaxel for previously treated, PD-L1-positive, advanced non-small-cell lung cancer (KEYNOTE-010): a randomised controlled trial. *Lancet Lond Engl.* (2016) 387:1540–50. 10.1016/S0140-6736(15)01281-7 26712084

[5] Pinal-Fernandez I Selva-O’ Callaghan A Grau JM. Diagnosis and classification of eosinophilic fasciitis. *Autoimmun Rev.* (2014) 13:379–82. 24424187 10.1016/j.autrev.2014.01.019

[6] Zampeli E Zervas E. Eosinophilic fasciitis following checkpoint inhibitor therapy with pembrolizumab. *Mediterr J Rheumatol.* (2021) 32:376–7. 35128334 10.31138/mjr.32.4.376 PMC8802204

[7] Mendoza FA Bai R Kebede AG Jimenez SA. Severe eosinophilic fasciitis: comparison of treatment with D-penicillamine plus corticosteroids vs. corticosteroids alone. *Scand J Rheumatol.* (2016) 45:129–34. 10.3109/03009742.2015.1067713 26525956 PMC4775409

[8] Chan KK Magro C Shoushtari A Rudin C Rotemberg V Rossi A Eosinophilic fasciitis following checkpoint inhibitor therapy: four cases and a review of literature. *Oncologist.* (2019) [Online ahead of print]. 10.1634/theoncologist.2019-0508 PMC7011633 32043775

[9] Moulton SJ Kransdorf MJ Ginsburg WW Abril A Persellin S. Eosinophilic fasciitis: spectrum of MRI findings. *AJR Am J Roentgenol.* (2005) 184:975–8. 10.2214/ajr.184.3.01840975 15728627

[10] Desvignes-Engelbert A Saulière N Loeuille D Blum A Chary-Valckenaere I. From diagnosis to remission: place of MRI in eosinophilic fasciitis. *Clin Rheumatol.* (2010) 29:1461–4. 10.1007/s10067-010-1508-1 20532934

[11] Bisschop C de Heer EC Brouwers AH Hospers GAP Jalving M. Rational use of 18F-FDG PET/CT in patients with advanced cutaneous melanoma: a systematic review. *Crit Rev Oncol Hematol.* (2020) 153:103044. 10.1016/j.critrevonc.2020.103044 32673997

[12] Kim HJ Lee S-W Kim GJ Lee JH. Usefulness of FDG PET/CT in the diagnosis of eosinophilic fasciitis. *Clin Nucl Med.* (2014) 39:801–2. 10.1097/RLU.0000000000000260 24152641

[13] Cheriet S Chastan M Levesque H Marie I. Positron emission tomography in the diagnosis of eosinophilic fasciitis. *QJM Mon J Assoc Physicians.* (2011) 104:987–8. 10.1093/qjmed/hcq218 21081565

